# How the pandemic affected psychological research

**DOI:** 10.1098/rsos.241311

**Published:** 2024-11-20

**Authors:** Mario Gollwitzer, Stephan Nuding, Leonhard Schramm, Andreas Glöckner, Robert Gruber, Katharina V. Hajek, Jan A. Häusser, Roland Imhoff, Selma C. Rudert

**Affiliations:** ^1^ Department of Psychology, Ludwig-Maximilians-Universität, Munich, Germany; ^2^ Department of Psychology, Universität zu Köln, Cologne, Germany; ^3^ Department of Communication Psychology, Berlin University of the Arts, Berlin, Germany; ^4^ Department of Media and Communication, Ludwig-Maximilians-Universität, Munich, Germany; ^5^ Department of Psychology, Justus-Liebig-Universität, Giessen, Germany; ^6^ Department of Psychology, Johannes-Gutenberg-Universität, Mainz, Germany; ^7^ Department of Psychology, RPTU Kaiserslautern-Landau, Landau, Germany

**Keywords:** COVID-19 pandemic, meta-science, open science, research quality, psychology

## Abstract

In the wake of the COVID-19 pandemic, many journals swiftly changed their editorial policies and peer-review processes to accelerate the provision of knowledge about COVID-related issues to a wide audience. These changes may have favoured speed at the cost of accuracy and methodological rigour. In this study, we compare 100 COVID-related articles published in four major psychological journals between 2020 and 2022 with 100 non-COVID articles from the same journal issues and 100 pre-COVID articles published between 2017 and 2019. Articles were coded with regard to design features, sampling and recruitment features, and openness and transparency practices. Even though COVID research was, by and large, more ‘observational’ in nature and less experimentally controlled than non- or pre-COVID research, we found that COVID-related studies were more likely to use ‘stronger’ (i.e. more longitudinal and fewer cross-sectional) designs, larger samples, justify their sample sizes based on *a priori* power analysis, pre-register their hypotheses and analysis plans and make their data, materials and code openly available. Thus, COVID-related psychological research does not appear to be less rigorous in these regards than non-COVID research.

## 1. Introduction

After the outbreak of SARS-CoV-2 in early 2020, there was an urgent need for immediate and solid scientific answers to pandemic-related questions. This led to an unprecedented increase in scientific articles published on the COVID-19 pandemic and the epidemiological, immunological, healthcare-related, social, political and psychological questions surrounding it. On PubMed, the world’s most exhaustive citation and abstracts database for the biomedical and life sciences literature, the number of COVID-related scientific publications grew to 2500 within the first three months after the outbreak [1]. By the end of October 2020, over 125 000 articles on COVID-related topics had been published—about a quarter of them being posted on preprint servers such as medRxiv, SSRN or arXiv [2]. Despite the advantages of preprint publications (i.e. quick and open access), the skyrocketing number of preprints has also contributed to a growing concern about the quality of the research: as many preprint servers have no quality assurance procedure whatsoever, authors can post any research there, no matter how flawed it is. While this represents a general concern with preprint servers, it may have even worse consequences during a global pandemic [3–5]. As there was a strong demand from the media and the public for answers on how to navigate the pandemic, many results from preprints were immediately spread by news outlets. While thus meeting the immediate demand for information, this practice undoubtedly represented a challenge for scientific integrity.

To address these challenges, peer-reviewed scientific journals started implementing policies that aimed at achieving a compromise between speedy publication of COVID-related articles on the one hand and a sufficient quality control procedure (i.e. peer-review) on the other hand. Juggling these demands was a burden, given the large number of papers submitted to journals on COVID-related issues: the editorial office of JAMA, a prestigious journal consortium specialized on medicine and public health issues, received more than 11 000 submissions between January and June 2020; more than three times as during the same period in 2019 (e.g. [6]). As a consequence, many journals implemented editorial policies allowing a prioritized handling of COVID-related papers, an accelerated peer-review process and a speedy publication of this research with open access. With these changes, journals managed to reduce their turnaround times for COVID-related articles by 50% [7]. Some journals managed to reduce their median turnaround times for COVID-related papers to just 6 days [8].

Even during the pandemic, there was a growing concern among scientists that such policies may favour speed over accuracy [9]: manuscripts that would normally not take the hurdle may now be accepted for publication (and vice versa; see [10]). Systematic comparisons between COVID-related and non-COVID articles in biomedical journals confirm this notion (i.e. COVID-related articles were more likely to suffer from selection biases in randomized control trials, lack of representativeness in cohort studies, comparability issues in case-control studies, etc. [11,12]). However, other analyses suggest that neither the prevalence of statistical reporting errors nor the level of scrutiny in peer reviews differed systematically between COVID-related and non-COVID articles [13,14].

## 2. COVID-related articles in psychology journals

The pandemic affected not only the biomedical and life sciences but also the social and behavioural sciences. Given the unprecedented challenges societies around the globe were facing, important and pressing questions needed to be addressed: How do people assess their personal risk of being infected with the virus? What can we do to help them calibrate that risk? How can we increase and maintain large-scale compliance with ‘non-pharmaceutical’ measures to decelerate the spread of the virus? What can be done about the spread of misinformation on the Internet and on social media? What is the optimal communication strategy to inform the public about new developments, policies and consequences? And so forth (see [15–17]).

Responding to these challenges, van Bavel *et al*. [18] compiled a list of topics they argued should be relevant for psychological science to address during the pandemic. The authors suggest a (broad) categorization of these topics into six thematic clusters (cf. fig. 1 in [18]): (i) threat perception (including risk assessment as well as prejudice), (ii) social context (including social norms, inequality and polarization), (iii) science communication (including research on misinformation and conspiracy beliefs), (iv) aligning individual and collective interests (including research on cooperation and moral decision-making), (v) leadership (including issues related to social identity), and (vi) stress and coping (including social isolation, intimate relationships or mental health). They reviewed selected findings for each of these concepts and identified ‘… important gaps researchers should move quickly to fill in the coming weeks and months’ [18, p. 460]. The idea was to focus specifically on these issues, given their immediate relevance for COVID-related societal questions (see also [19,20]).

Of course, the pandemic-triggered changes in editorial policies that we have described above also affected psychological journals. Many journals installed a ‘fast track’ route to publication, with prioritized and accelerated turnaround times and open access publication (e.g. [21]; see also the special collection of COVID-related articles in *Psychological Science*: https://journals.sagepub.com/page/pss/covid-19).

Did these policy changes in psychological journals lower the quality threshold for published articles? And were COVID-related articles affected more strongly by these changes than non-COVID-related articles? This article addresses these questions. More specifically, we analysed whether COVID-related articles in psychology journals (that adopted fast-track policies in 2020/2021) differed from non-COVID articles with regard to (i) their design (e.g. experimental/observational, cross-sectional/longitudinal, measured/manipulated IV, etc.) with implications for the study’s *internal validity*, (ii) their sampling and recruitment strategy (including sample sizes as well as demographic heterogeneity of the samples) with implications for the study’s *external validity*, and (iii) their adoption of contemporary standards of openness and transparency (including pre-registered hypotheses and analysis plans, open data, open material, open code, etc.), which is relevant in order to assess a study’s *reproducibility*. In addition, we inspected authorships and collaborative networks between psychological researchers.

Our research is looking at how the urgent need for scientific knowledge during the pandemic and the consequential policy changes in the publication system—rapid review processes and incentives for designing, conducting and analysing studies in a very short time frame—may have affected the level of methodological rigour and the quality of psychological science. While the COVID pandemic represents a specific case, we think that our findings are informative even beyond this context: more generally speaking, we investigate how resilient current methodological standards in psychological science—in particular, standards regarding validity, robustness, openness and transparency—are when systemic pressures (such as incentives that favour speed over accuracy) are present. If COVID-related research were, on average, weaker in terms of internal and/or external validity than non-COVID research or if non-COVID research adhered more strictly to openness and transparency standards than COVID-related research, this would imply a certain volatility of methodological standards and a systemic challenge for the reform movement in psychological science.

## 3. Method

We decided to randomly select 100 COVID-related articles (henceforth ‘Category A’) published between 2020 and 2022 in four major psychological journals, two of them focusing on social/personality psychology (*Social Psychological and Personality Science* (SPPS) and *Personality and Social Psychology Bulletin* (PSPB)) and two covering a broader range of psychological topics (*Journal of Applied Psychology* (JAP) and *Psychological Science* (PS)). Each selected COVID-related article was matched with a non-COVID article (Category B) from the same issue of the same journal—in most cases, the article that immediately followed or preceded the COVID-related article in the issue where it was published. Again, the target number for Category B articles was 100. Finally, we selected a third batch of articles (Category C) from the same journals, published 3 years before the pandemic (i.e. between 2017 and 2019), but on the same or a closely related topic as the respective Category B article. The reason for doing so was to analyse whether the pandemic-related changes in editorial policies and peer-review practices (see [12]) affected not only COVID-related but also non-COVID articles. Lockdown measures in various countries often led to research laboratories being shut down for more than a year, with drastic consequences, especially for early-career researchers (e.g. [22]). Conducting research with comparably less rigorous designs (e.g. quasi-experimental instead of experimental studies) or submitting manuscripts that had been lying in one’s file drawer for a while may have been the only way to remain scientifically ‘productive’ during the COVID pandemic for many. If this were the case, we should witness systematic differences between non-COVID articles published in 2020–2022 and those published in 2017–2019.

### 3.1. Article selection

We started by identifying all Category A articles using the target journals’ search engines and the keywords ‘covid’, ‘corona’ or ‘pandemic’ included in the title, keywords and/or abstract and a publication date between 1 January 2020 and 31 May 2022. We filtered for ‘research article’ or ‘brief report’ (SPPS, PS and PSPB) and ‘journal article’ (JAP) as the article type. Next, we selected a corresponding Category B article for each Category A article. We chose the first empirical non-COVID-related articles in the same issue and, if possible, from the same section within it (e.g. a section of a special issue, general research articles or featured articles). If the COVID-related article was an online first publication, we took the subsequent empirical non-COVID-related publication from the journal’s list of online publications on the day of the search process. For Category C, we selected articles that were published 3 years before the respective Category A or B article. If Category A and B articles were already published in a journal, we selected the first empirical article in the same journal but in an issue 3 years before.

We reviewed whether each article covered empirical research and has been correctly assigned to the categories. The initial search was conducted in June 2022 and a final check on 3 November 2022. In total, 119 Category A articles met the inclusion criteria, which resulted in 357 articles in total. We used 15 articles for a pilot phase and excluded them from the coding. The list of all articles can be retrieved from the OSF project (https://osf.io/2b5t4/).

### 3.2. Features and coding manual

We identified relevant features associated with an article’s quality and defined them and their indicators in a coding manual to achieve high interrater reliability between all coders. We pretested the manual using 15 articles (five per category), refined the manual and created a coding solution for nine articles (three per category), which were used as training articles for the coders. We pilot-tested the manual with six coders. They coded the training articles and gave feedback on the manual and coding procedure.

Afterwards, we calculated the overall coders’ interrater agreement (Fleiss’ Kappa, intraclass correlation coefficient (ICC)), including the coding solution. We further clarified the manual to prevent systematic coding errors. Additionally, we instructed the coders to be strict and code only when the information in the articles met the manual descriptions exactly. Coders could comment and discuss it with a project member if they were unsure. We provide a summary of each feature below. The coding manual with detailed descriptions and all indicators can be retrieved from the OSF project. We standardized the coding procedure by describing formal aspects in the manual (e.g. each study in an article has to be coded unless stated otherwise).

#### 3.2.1. General features

These include the date of publication (online and issue) and a category check whether categories A and B articles were correctly assigned. We coded the research topic (*κ* = 0.52) using the list of six thematic clusters as suggested by van Bavel *et al*. [18]. Other topics could be coded using a free text field. We also coded the number of authors (ICC = 1.00), the number of different affiliations (ICC = 0.94), the number of reported studies (ICC = 1.00), the number of references (ICC = 0.96) and the word count of the articles (ICC = 0.90). Regarding hypothesis testing, we coded whether the main research question was exploratory, confirmatory or unclear (κ = 0.35), and whether the main hypotheses were confirmed, partly confirmed, not confirmed, unclear or whether this feature was not applicable when the research question was exploratory (*κ* = 0.46).

#### 3.2.2. Design features

These include whether and how the independent variable (IV) was manipulated (i.e. experimentally manipulated between-subjects, manipulated within-subjects, not manipulated or unclear; *κ* = 0.47) and, in the latter cases, how the IV was measured (e.g. self-report questionnaires, behavioural outcome measures, tests, biological or physiological measures, other or unclear; *κ* = 0.35). We coded how the dependent variable (DV) was measured (*κ* = 0.58) in the same way and whether the design (*κ* = 0.95) was cross-sectional (e.g. survey or experiment with a single occasion of measurement), a longitudinal panel study with the same participants or a repeated cross-sectional study with several measurement occasions but different participants.

#### 3.2.3. Sampling and recruitment

Regarding the sample and recruitment strategy, we coded the sample size (ICC = 0.95) after data exclusion, gender proportion (ICC_female_ = 0.98, ICC_male_ = 0.97 and ICC_other_ = 1.00), sample age (ICC_mean_ = 1.00 and ICC_standard_deviation_ = 1.00), recruitment strategy (i.e. student sample and use of paid crowdsourcing platforms such as MTurk, Prolific, etc., other or unclear; *κ* = 0.82) and the study environment in which the data were collected (i.e. online, laboratory or field study; *κ* = 0.86).

#### 3.2.4. Open science

Features that relate to the adaptation of contemporary standards of openness and transparency include whether open data (*κ* = 0.58), open material (*κ* = 0.60) and open code (*κ* = 0.33) were available for the studies of an article, whether existing data were reanalysed and whether the article referred to a registered report or a pre-registration (*κ* = 0.69). Furthermore, we coded whether and how the studies’ sample sizes were determined (e.g. *a priori* power analysis, resource-based or other justifications or no justifications; *κ* = 0.67).

### 3.3. Coding

The target number was 100 articles per category (i.e. A, B and C). This number was determined based on feasibility, not based on *a priori* power analysis (because we were not able to determine a population effect size for our statistical tests *a priori*). A total of 300 articles would provide us with sufficient power to detect a small- to medium-size effect (for instance, a *χ*²-test with four coded feature categories and, thus, (4 – 1) × (3 – 1) = 6 degrees of freedom, would have a 90% power to detect an effect of *ω* = 0.24 on a 5% significance level with a sample of *n* = 300 articles). The rating was conducted between 29 November 2022 and 9 May 2023. One coder coded each article. Each coder was trained by introducing them to the coding manual and applying it to nine training articles (for which the correct solutions were provided afterwards). In total, 12 coders rated on average *M* = 25.5 articles (s.d. = 11.9, range = 8; 51). The average coding time per article was 38 min (s.d. = 13 min). Each coder received an individual allocation of randomly drawn articles. We ensured that for each Category A article, the respective Category B and C articles were coded and that each coder rated a comparable number of articles in each of the three categories.

As one can see from the interrater agreement statistics provided above, the lowest values for Fleiss’ Kappa were obtained for deciding (i) whether or not the analysis code was available, (ii) whether the research question was confirmatory, exploratory, etc., and (iii) how the IV was operationalized. In these three cases, the agreement between coders was ‘fair’ at best (according to [23]). In six other cases (confirmation status, operationalization of the IV and the DV, research topic/cluster, open data and open materials), the interrater agreement was ‘moderate’, and in all other cases, it was at least ‘substantial’ (for critical discussions of Landis and Koch’s taxonomy, see [24,25]).

### 3.4. Data preparation

All changes to the initial coding (e.g. correction of input errors) were made in R and can be reproduced using the R scripts (see OSF). Cases in which more than one coding criterion applied within a feature (e.g. when a study sample consisted of students as well as paid crowdsourced participants) were assigned to a separate category (‘multiple codings’).

## 4. Results

We only present the main findings here. More detailed tables with all relative frequencies can be found in the OSF project (https://osf.io/2b5t4) in the electronic supplementary material.

### 4.1. General features

#### 4.1.1. Research topics

All 169 COVID-related studies that belonged to Category A were categorized into the six thematic clusters suggested by van Bavel *et al*. [18]. One hundred and fifty-seven of them (i.e. 93%) could be assigned to a cluster; only 7% could not. Seventeen studies (i.e. 10%) could not be clearly categorized into a cluster. Most studies could be classified into the ‘stress and coping’ cluster (27%) and the ‘aligning individual/collective interests’ cluster (20%); fewer studies dealt with ‘social context’ (15%) or ‘threat perception’ (13%), and the smallest clusters were ‘science communication’ (5%) and ‘leadership’ (2%).

#### 4.1.2. Hypothesis testing

Here, we found no differences between COVID-related (confirmatory: 70.4%, exploratory: 18.9%, unclear: 4.7% and multiple codings: 5.9%), non-COVID (confirmatory: 70.3%, exploratory: 16.0%, unclear: 9.1% and multiple codings: 4.6%) and pre-COVID (confirmatory: 59.5%, exploratory: 26.5%, unclear: 8.8% and multiple codings: 5.1%) studies, *χ*²(6) = 11.76, *p* = 0.067, *ω* = 0.14.

#### 4.1.3. Hypotheses confirmation

Regarding the prevalence of hypothesis-confirming findings of confirmatory research, we did not find any meaningful differences between our three article categories: COVID-related (confirmed: 40.8%, not confirmed: 4.1%, partially confirmed: 26.0% and other/unclear: 29.0%), non-COVID (confirmed: 49.3%, not confirmed: 2.7%, partially confirmed: 23.3% and other/unclear: 24.7%) and pre-COVID (confirmed: 44.2%, not confirmed: 4.7%, partially confirmed: 18.6% and other/unclear: 32.6%) studies, *χ*²(6) = 7.52, *p* = 0.276, *ω* = 0.11. In total, 272 confirmed main hypotheses (i.e. 45.1% of all hypotheses) were reported in the 603 studies.

#### 4.1.4. Other general features

While COVID-related articles had larger ranges regarding the number of authors (from 1 to 101) and affiliations (from 1 to 62) than non-COVID articles (1–36 authors and 1–36 affiliations), the variances were not significantly different, neither for authors, *F*_1,386_ = 1.77, *p* = 0.185, nor for affiliations, *F*_1,386_ = 0.59, *p* = 0.442. Two COVID-related articles and six non-COVID articles were written by particularly large (i.e. two standard deviations above the mean) consortia of authors (15 or 101 authors for the COVID articles and between 14 and 36 for the non-COVID articles). Non-COVID articles had a larger range regarding the number of authors and affiliations than pre-COVID articles (1–10 authors and 1–8 affiliations); yet, again, variances were not significantly different, neither for authors, *F*_1,432_ = 0.25, *p* = 0.618, nor for affiliations, *F*_1,432_ = 0.01, *p* = 0.930. The variances of COVID and pre-COVID articles were not significantly different as well, neither for authors, *F*_1,382_ = 2.66, *p* = 0.104, nor for affiliations, *F*_1,382_ = 0.92, *p* = 0.339.

The article length of the three categories differed, *F*_2,596_ = 16.64; *p* < 0.001, *η*² = 0.05: COVID-related articles were, on average, shorter (*M* = 6868 words; s.d. = 2644 words) than non-COVID (*M* = 8839 words; s.d. = 4023 words), *t*(377.06) = −5.80, *p* < 0.001, *d* = −0.56 [CI_95_ = −0.77; −0.36] and pre-COVID articles (*M* = 8663 words; s.d. = 3824 words), *t*(370.51) = −5.39, *p* < 0.001, *d* = −0.54 [CI_95_ = −0.74; −0.33]. Non-COVID und pre-COVID articles did not differ, *t*(428) = 0.47, *p* = 0.642, *d* = 0.04 [CI_95_ = −0.14; 0.23].

### 4.2. Design features

#### 4.2.1. Design

The majority of studies reported a cross-sectional design (COVID: 61.5%, non-COVID: 77.2% and pre-COVID: 77.2%). Yet, the number of longitudinal panel designs (i.e. same participants and multiple measurement occasions) was larger for COVID-related (32.0%) compared with non-COVID (19.6%) and pre-COVID (19.1%) studies, and the remaining studies reported a repeated cross-sectional design or could not be clearly categorized (COVID: 6.5%, non-COVID: 3.2% and pre-COVID: 3.7%), *χ*²(4) = 15.24, *p* = 0.004, *ω* = 0.16.

The number of studies with experimentally manipulated IVs (versus measured or manipulated within-participants) was smaller for COVID-related studies (manipulated: 21.3%, measured: 71.6%, within: 5.3% and other/unclear: 1.8%) compared with non-COVID (manipulated: 34.7%, measured: 48.4%, within: 14.6% and other/unclear: 2.3%) or pre-COVID (manipulated: 28.4%, measured: 57.2%, within: 9.8% and other/unclear: 4.7%) studies, *χ*²(6) = 26.14, *p* < 0.001, *ω* = 0.21. Thus, in most COVID-related studies, the IV was measured rather than manipulated.

#### 4.2.2. Type of data

**Figure 1.**
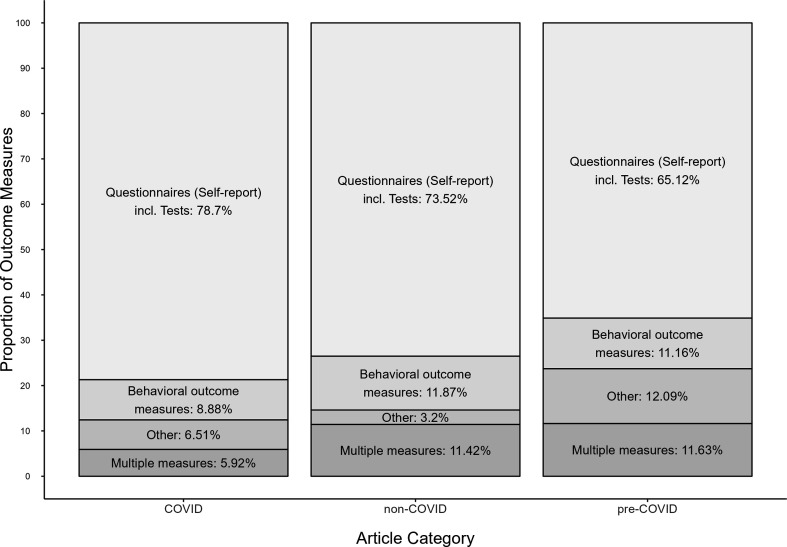
Types of data for the dependent variable (DV) in COVID-related (*k* = 169) studies, non-COVID (*k* = 219) studies and pre-COVID (*k* = 215) studies.

The majority of COVID-related studies used self-report measures for both the IV (52.7%) and the DV (78.7%). Self-report measures were less often used in non-COVID (IV: 39.3% and DV: 73.5%) and pre-COVID articles (IV: 40.9% and DV: 65.1%), *χ*²(2) = 7.94, *p* = 0.019, *ω* = 0.11.^1^ Regarding the DV (i.e. outcome measures), COVID-related articles were more likely to rely on self-report measures and less likely to rely on behavioural outcomes compared with non- or pre-COVID articles (see figure 1). Here, types of data (coded as self-report, behavioural outcomes, other or multiple) were unequally distributed across the three article categories, *χ*²(6) = 19.19, *p* = 0.004, *ω* = 0.18.

### 4.3. Sampling and recruitment

#### 4.3.1. Sample sizes

**Figure 2.**
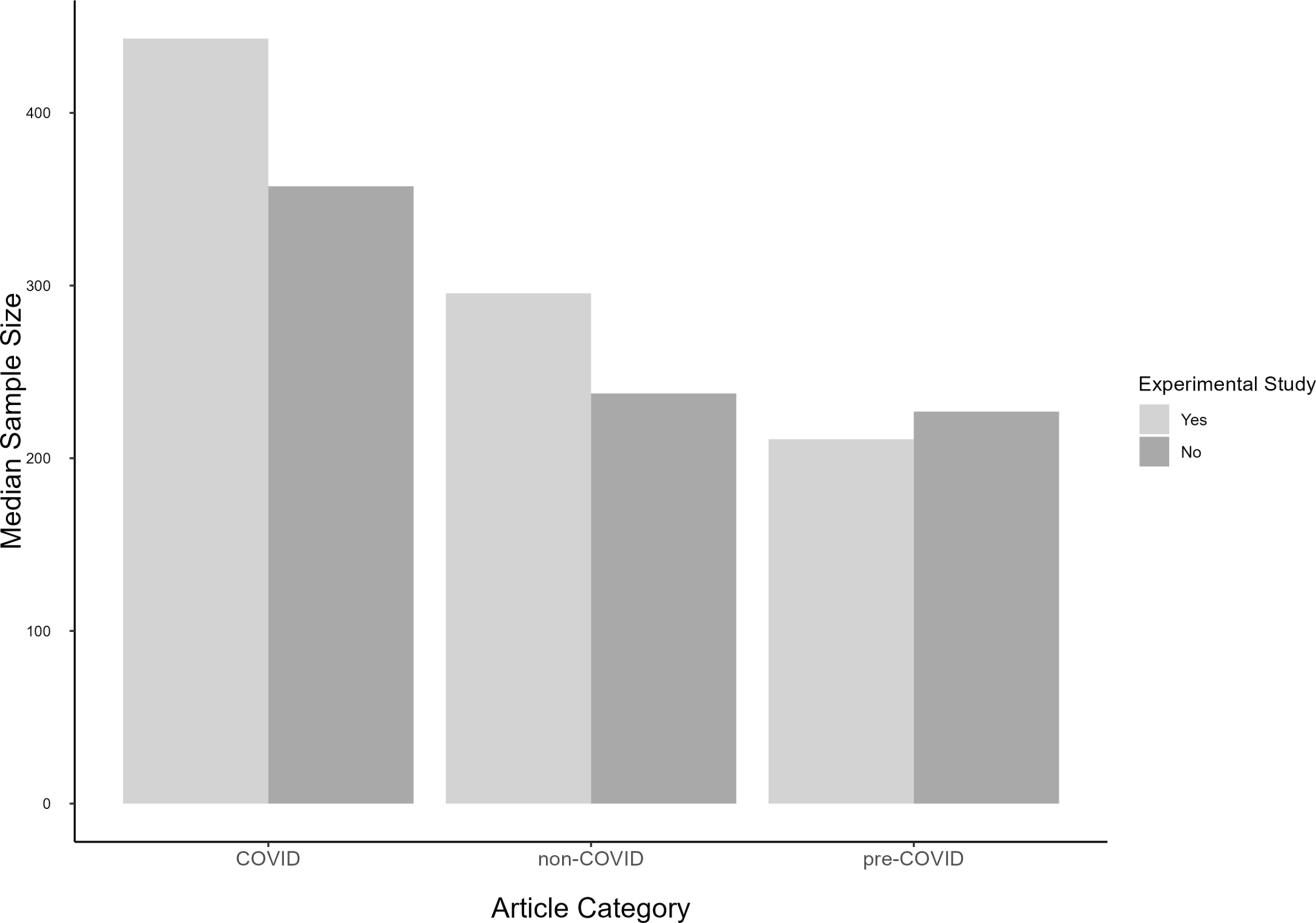
Median sample sizes in COVID-related (*k* = 169, of which 36 were experimental) studies, non-COVID (*k* = 217, of which 76 were experimental) studies and pre-COVID (*k* = 211, of which 61 were experimental) studies.

Given a small number of studies with extremely large sample sizes, we used the median sample size instead of the mean sample size. Median sample sizes differed significantly across article categories, *H*(2) = 24.00, *p* < 0.001, *η*² = 0.04: COVID-related articles had significantly larger sample sizes (*Mdn* = 383.0) than non-COVID (*Mdn* = 250.5), *H*(1) = 12.91, *p* < 0.001, *η*² = 0.03 or pre-COVID (*Mdn* = 222.0) articles, *H*(1) = 21.82, *p* < 0.001, *η*² = 0.06, while non-COVID and pre-COVID articles did not differ with regard to their sample sizes, *H*(1) = 2.62, *p* = 0.105, *η*² < 0.01. This pattern applies also to experimental studies (see figure 2), *H*(2) = 15.13, *p* < 0.001, *η*² = 0.08.

#### 4.3.2. Demographics

First, we looked at the gender distributions in each study and tested whether the average (i.e. median) relative frequency of women (as an arbitrary reference category) differed across the three article categories. This was not the case: the median frequency did not differ significantly between categories (COVID: 53.9, non-COVID: 52.0 and pre-COVID: 53.0), *H*(2) = 3.95, *p* = 0.139, *η*² < 0.01. Second, we looked at age distributions in each study; we found significant differences regarding participants’ average ages (COVID: 37.29, non-COVID: 32.52 and pre-COVID: 31.17), *F*_2,472_ = 19.72; *p* < 0.001, *η*² = 0.08 and the reported standard deviations of age (COVID: 10.90, non-COVID: 8.82 and pre-COVID: 8.36), *F*_2,397_ = 12.30; *p* < 0.001, *η*² = 0.06 between the three categories.

#### 4.3.3. Recruitment

The majority of COVID-related studies (52.7%) worked with paid participants recruited via crowdsourcing platforms (e.g. MTurk and Prolific). Non-COVID studies used relatively fewer crowdsourced samples (36.5%), and this number was even lower for pre-COVID studies (32.1%). By contrast, the number of student samples was smaller for COVID-related studies (6.5%) than for non-COVID (25.1%) or pre-COVID studies (25.6%). The remaining studies used other or multiple recruitment strategies or could not be clearly categorized (COVID: 40.8%, non-COVID: 38.4% and pre-COVID: 42.3%). The difference in recruitment strategies between the three article categories was significant and quite large, *χ*²(4) = 32.85, *p* < 0.001, *ω* = 0.23.

#### 4.3.4. Data collection environment

The majority of COVID-related studies (76.3%) used data that were collected online. This number was smaller for non-COVID studies (62.1%) and pre-COVID studies (53.0%). Yet, as we will discuss later, this may be due to the fact that data collected for non-COVID studies were collected before research facilities were closed due to lockdowns. That said, the number of studies in which data were collected in the field were comparable across the three categories (COVID: 16.0%, non-COVID: 16.9% and pre-COVID: 17.7%), but the number of studies that used data collected in the laboratory decreased after the outburst of the pandemic (COVID: 3.0%, non-COVID: 11.4% and pre-COVID: 20.0%). The number of studies that collected data in multiple different environments or that could not be classified based on the information in the respective article was smaller for COVID-related studies (COVID: 4.7%, non-COVID: 9.6% and pre-COVID: 9.3%). Overall, data collection environment varied significantly between the three article categories, *χ*²(6) = 34.54, *p* < 0.001, *ω* = 0.24.

### 4.4. Open science

#### 4.4.1. Availability of data, materials and code

Sharing data was more frequent for COVID (52.6%) and non-COVID (48.9%) compared with pre-COVID articles (16.3%), while the remaining articles either did not say anything about the data being available (COVID: 37.9%, non-COVID: 45.2% and pre-COVID: 74.4%) or re-used existing datasets (COVID: 9.5%, non-COVID: 5.9% and pre-COVID: 9.3%), *χ*²(4) = 72.81, *p* < 0.001, *ω* = 0.35. Similar results were found for sharing materials (COVID: 71.6%, non-COVID: 41.6% and pre-COVID: 20.5%), *χ*²(2) = 101.38, *p* < 0.001, *ω* = 0.41 and for sharing code (COVID: 52.1%, non-COVID: 41.6% and pre-COVID: 11.2%), *χ*²(2) = 80.49, *p* < 0.001, *ω* = 0.37. In sum, COVID-related articles (and, to some extent, also non-COVID articles) were more likely to share their data, materials and code compared with pre-COVID articles.

#### 4.4.2. Pre-registration

Pre-registrations (of hypotheses, materials and analytic procedures) were much more common for COVID-related studies (32.5% including registered reports) than for non-COVID studies (11.0%) or pre-COVID studies (7.4%). However, these numbers also show that the majority of studies in each category did not refer to any pre-registration document (COVID: 67.5%, non-COVID: 89.0% and pre-COVID: 92.6%), χ²(2) = 50.88, *p* < 0.001, *ω* = 0.29.

#### 4.4.3. Sample size determination

**Figure 3.**
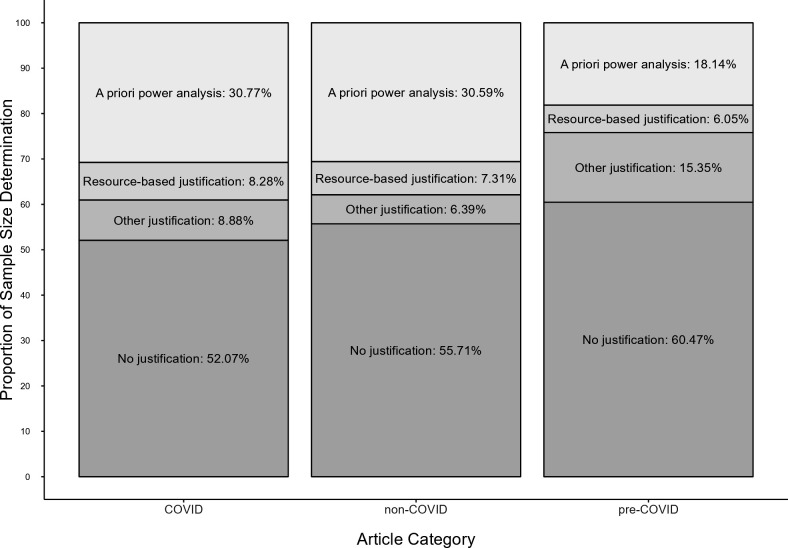
Sample size justifications in COVID-related (*k* = 169) studies, non-COVID (*k* = 219) studies and pre-COVID (*k* = 215) studies.

About half of the studies coded in each category did not justify their sample sizes (COVID: 52.1%, non-COVID: 55.7% and pre-COVID: 60.5%). That said, the number of studies in which sample sizes were determined based on *a priori* power analysis was much higher for COVID (30.8%) and non-COVID (30.6%) compared with pre-COVID studies (18.1%), and the remaining studies claimed that resources (e.g. time and financial) or other factors limited their sample sizes (COVID: 17.2%, non-COVID: 13.7% and pre-COVID: 21.4%), *χ*²(4) = 13.20, *p* = 0.010, *ω* = 0.15 (figure 3). More details, including differences between social or personality psychology journals and journals with a broader research focus, can be found in the electronic supplementary material.

## 5. Discussion

Is there reason to believe that COVID-related psychological research is of lower quality than non-COVID or pre-COVID research published in psychological journals? According to the findings presented here, the answer is no. Contrary to what one might expect given the restricted conditions for conducting research and the accelerated peer-review system during the pandemic, COVID-related studies (in comparison with non-COVID or pre-COVID studies) were actually *more* likely to (i) use ‘stronger’ (i.e. more longitudinal and fewer cross-sectional) designs, (ii) use larger samples, (iii) justify their sample sizes based on *a priori* power analysis, (iv) pre-register their hypotheses and analysis plans, and (v) make their data, materials and code openly available. At the same time, COVID-related studies were no less likely to (i) be confirmatory in nature, (ii) use field data, or (iii) rely on more selective samples than non- and pre-COVID studies. That said, COVID-related studies were more likely to (i) solicit their data via online surveys, (ii) rely on self-report data, and (iii) use non-experimental designs. Thus, COVID research was, by and large, more ‘observational’ in nature and less experimentally controlled than non- or pre-COVID research. This may have to do with closed research facilities (i.e. laboratory studies were difficult or even impossible to conduct in large parts of the world) as well as with the research questions addressed by COVID-related research.

Most of this research dealt with stress/coping and with aligning individual versus collective interests. Stress and coping were highly important and pressing issues during the pandemic, given the rapidly changing epidemiological situation, the uncertainty about how to decelerate the spread of the virus, instances of psychological losses and the lack of control and foreseeability. Research dealing with the alignment of individual versus collective interests (i.e. research on the social dilemma nature of the pandemic situation; e.g. [17,26,27]) was necessary to understand the conditions under which people would accept non-pharmaceutical measures (such as lockdowns), would trust their institutions and would show pro-social, cooperative behaviour toward others in times of crisis.

While concerns about low-quality research making it into high-impact journals due to accelerated research procedures and speedy peer-review processes are valid and important, there is apparently no reason to be alarmed when it comes to psychological research on COVID-related issues. At least on those quality indicators that we coded and at least for the studies we selected here, we did not witness any significant loss of quality. That said, one might, of course, argue that ‘quality’ entails much more than what we coded here. Additional aspects, such as the quality of the data analysis, the prevalence of statistical errors, the reproducibility of the findings, the psychometric properties of the materials that were used, etc., were not coded here (due to resource constraints on our side but also due to information that we cannot access). Another limitation that should be noted is that we only focused on four specific journals, two of them specialized on social psychology and personality research. Other areas and journals in which psychological studies related to the COVID pandemic were frequently published (such as health psychology, clinical psychology and educational psychology) were not addressed here, and things may look different there. So there may well be detectable and objectifiable speed–accuracy trade-offs that we did not observe. To gain a more comprehensive picture of the quality of COVID-related research (in psychology and beyond), a more thorough and broader inspection of the published research is necessary (for example, see [11]).

Our decision to compare COVID-related articles with non-COVID articles and to compare non-COVID articles with pre-COVID articles was made with the intention to maximize comparability and to increase the internal validity of our own design. However, the internal validity was certainly not optimal: Even though the non-COVID articles in ‘Category B’ were published between 2020 and 2022 and the pre-COVID articles in ‘Category C’ were published between 2017 and 2019, it is quite possible and likely that the studies reported in these articles were conducted quite some time before they were published: Category B articles may have used data that had been lying in the file drawer for a while (which may be due to laboratory shutdowns, as explained earlier). Therefore, it is difficult to interpret differences between Category B and C articles; more specifically, we cannot be sure whether the higher rigorousness of COVID-related research compared with non- and pre-COVID research, as described earlier, points to higher research standards (e.g. regarding sample size justifications, openness and transparency) in general or rather to higher standards specifically applied to COVID-related topics. However, in any case, our analyses illustrate that altered research practices and publication routines did not erode research quality and transparency.

Our findings suggest that COVID-related psychological research ticked at least as many ‘quality boxes’ as non-COVID or pre-COVID research. It is a good sign that psychological research during a time of crisis, when answers to many societal questions were direly needed, did not lower its standards even though the review system might have favoured speed over accuracy during this time. This also suggests that the increase in methodological rigour in psychological science, which has mainly been triggered by the ‘replication crisis’ and the reform movements that this crisis has released, is quite robust against systemic challenges such as the (internal and external) pressure to prioritize speed in the dissemination of scientific knowledge. There is hope that the current methodological standards that represent our field are sufficiently resilient against changes in the scientific incentive system.

## Data Availability

Raw data and materials that are necessary to reproduce our results as well as additional supplementary materials are available on the OSF platform [28].

## Footnotes

^1^
This test looks at the frequency distributions regarding how the IV was operationalized (i.e. self-report measure versus manipulated/other measure) across the three article categories.
