# Correction to: ‘How the pandemic affected psychological research’

**DOI:** 10.1098/rsos.242064

**Published:** 2024-12-11

**Authors:** Mario Gollwitzer, Stephan Nuding, Leonhard Schramm, Andreas Glöckner, Robert Gruber, Katharina V. Hajek, Jan A. Häusser, Roland Imhoff, Selma C. Rudert

**Affiliations:** ^1^ Department of Psychology, Ludwig-Maximilians-Universität, Munich, Germany; ^2^ Department of Psychology, Universität zu Köln, Cologne, Germany; ^3^ Department of Communication Psychology, Berlin University of the Arts, Berlin, Germany; ^4^ Department of Media and Communication, Ludwig-Maximilians-Universität, Munich, Germany; ^5^ Department of Psychology, Justus-Liebig-Universität, Giessen, Germany; ^6^ Department of Psychology, Johannes-Gutenberg-Universität, Mainz, Germany; ^7^ Department of Psychology, RPTU Kaiserslautern-Landau, Landau, Germany

**Keywords:** COVID-19 pandemic, meta-science, open science, research quality, psychology

*R. Soc. Open Sci*. 11, 241311 (Published online 20 November 2024). (https://doi.org/10.1098/rsos.241311).

Upon re-reading our article (Gollwitzer, M., Nuding, S., Schramm, L., Glöckner, A., Gruber, R., Hajek, K. V., Häusser, J. A., Imhoff, R., & Rudert, S. C. (2024). How the pandemic affected psychological research. *Royal Society Open Science, 11*, 241311. https://doi.org/10.1098/rsos.241311) after it was published, we spotted some minor errors in Section 4.1.4 (‘Other general features’).

More specifically, the degrees of freedom for the *F*-statistics we reported there as well as the *F* values and descriptive statistics are not correct. What follows now is a corrected version of the respective paragraphs:

[...]

## 4.1.4. Other general features

While COVID-related articles had larger ranges regarding the number of authors (from 1 to 101) and affiliations (from 1 to 62) than non-COVID articles (1−36 authors and 1−36 affiliations), the variances were not significantly different, neither for authors, *F*(1,198) = 0.23, *p* = 0.636, nor for affiliations, *F*(1,198) = 0.03, *p* = 0.868. One COVID-related article and three non-COVID articles were written by particularly large (i.e. two standard deviations above the mean) consortia of authors (101 authors for the COVID article and between 18 and 36 for the non-COVID articles). Non-COVID articles had a larger range regarding the number of authors and affiliations than pre-COVID articles (1−10 authors and 1−8 affiliations); yet, again, variances were not significantly different neither for authors, *F*(1,198) = 3.05, *p* = 0.082, nor for affiliations, *F*(1,198) = 2.02, *p* = 0.157. The variances of COVID and pre-COVID articles were not significantly different as well, neither for authors, *F*(1,198) = 1.65, *p* = 0.201, nor for affiliations, *F*(1,198) = 1.31, *p* = 0.254.

The article length^1^ of the three categories differed, *F*(2,295) = 6.41; *p* = 0.002, *η*² = 0.04: COVID-related articles were, on average, shorter (*M* = 6873 words; s.d. = 3059 words) than non-COVID (*M* = 8611 words; s.d. = 3956 words), *t*(186.22) = −3.48, *p* = 0.001, *d* = −0.49 [CI95 = −0.77; −0.21] and pre-COVID articles (*M* = 8204 words; s.d. = 3690 words), *t*(188.09) = −2.76, *p* = 0.006, *d* = −0.39 [CI95 = −0.68; −0.11]. Non-COVID und pre-COVID articles did not differ, *t*(196) = 0.75, *p* = 0.455, *d* = 0.11 [CI95 = −0.17; 0.39].

[...]

Most importantly, the pattern of results does not change: Effects that were statistically significant in the published version are still significant in the corrected version, and vice versa. Therefore, the interpretation of these findings and the discussion of the results do not need to be changed. It is just the numbers in that particular paragraph that need to be corrected.

In addition, we also noticed that may not have been entirely clear how many articles per category were (a) identified, (b) used for piloting, and (c) used for the final analyses. As stated in Section 3.1, we identified 357 articles in total. Fifteen of those were used for piloting. From the remaining 342 articles, 100 COVID-related articles (i.e., Category A) were randomly drawn and matched with 100 non-COVID articles (i.e., Category B) and 100 pre-COVID articles (i.e., Category C), which resulted in 300 coded articles in total.

## Footnotes

^1^
No word count could be retrieved for two articles.

